# Calcineurin A Is Essential in the Regulation of Asexual Development, Stress Responses and Pathogenesis in *Talaromyces marneffei*

**DOI:** 10.3389/fmicb.2019.03094

**Published:** 2020-01-21

**Authors:** Yan-Qing Zheng, Kai-Su Pan, Jean-Paul Latgé, Alex Andrianopoulos, Hong Luo, Ru-Fan Yan, Jin-Ying Wei, Chun-Yang Huang, Cun-Wei Cao

**Affiliations:** ^1^Department of Dermatology and Venereology, The First Affiliated Hospital of Guangxi Medical University, Nanning, China; ^2^School of Medicine, University of Crete, Heraklion, Greece; ^3^School of Biosciences, The University of Melbourne, Parkville, VIC, Australia

**Keywords:** *Talaromyces* (*Penicillium*) *marneffei*, calcineurin, morphogenesis, cell wall integrity, immune escape, virulence

## Abstract

*Talaromyces marneffei* is a common cause of infection in immunocompromised patients in Southeast Asia and Southern China. The pathogenicity of *T. marneffei* depends on the ability of the fungus to survive the cytotoxic processes of the host immune system and grow inside host macrophages. These mechanisms that allow *T. marneffei* to survive macrophage-induced death are poorly understood. In this study, we examined the role of a calcineurin homolog (*cnaA*) from *T. marneffei* during growth, morphogenesis and infection. Deletion of the *cnaA* gene in *T. marneffei* resulted in a strain with significant defects in conidiation, germination, morphogenesis, cell wall integrity, and resistance to various stressors. The Δ*cnaA* mutant showed a lower minimal inhibitory concentration (MIC) against caspofungin (16 μg/ml to 2 μg/ml) and micafungin (from 32 μg/ml to 4 μg/ml) compared with the wild-type. These results suggest that targeting calcineurin in combination with echinocandin treatment may be effective for life-threatening systemic *T. marneffei* infection. Importantly, the *cnaA* mutant was incapable of adapting to the macrophage environment *in vitro* and displayed virulence defects in a mouse model of invasive talaromycosis. For the first time, a role has been shown for *cnaA* in the morphology and pathogenicity of a dimorphic pathogenic filamentous fungus.

## Introduction

Calcineurin is a Ca^2+^/calmodulin (CaM)-dependent protein phosphatase that is ubiquitous and conserved among eukaryotes. The heterodimeric calcineurin protein consists of a catalytic subunit (A) that binds to the calcium sensor CaM and a regulatory subunit (B) that contains four Ca^2+^-binding domains. The functions of calcineurin have been studied in a variety of fungal species, and it plays important roles in the regulation of cation homeostasis, morphogenesis, cell wall integrity, and pathogenesis (Rusnak and Mertz, 2000; Fox et al., 2001; Fox and Heitman, 2002). In filamentous fungi, calcineurin regulates conidial architecture, polarized growth extension and branching, sclerotial and appresorial development, cell wall integrity and stress adaptation (Fortwendel et al., 2009; Juvvadi et al., 2014; Juvvadi and Steinbach, 2015). Calcineurin activation leads to the dephosphorylation and activation of the transcription factor Crz1p/Tcn1p, which is involved in cell survival and calcium homeostasis in *Saccharomyces cerevisiae* (Cyert, 2003; Roque et al., 2016). It is involved in antifungal tolerance, cell morphogenesis (Sanglard et al., 2003; Bader et al., 2006; Cordeiro Rde et al., 2014), growth in an alkaline pH or high-temperature environment, membrane stress, mating, and virulence in *Candida albicans* (Cruz et al., 2002; Reedy et al., 2010; Liu et al., 2014). Previous reports on the dimorphic fungus *Paracoccidioides brasiliensis* have implicated calcineurin in morphogenesis, environmental stress responses and mycelium-to-yeast dimorphism (Fernandes et al., 2005; Campos et al., 2008; Matos et al., 2013).

*Talaromyces marneffei* is an emerging opportunistic fungal pathogen that is endemic in southern China, Taiwan, Hong Kong, Thailand, Laos, Vietnam, and northeastern India (Supparatpinyo et al., 1994; Antinori et al., 2006; Vanittanakom et al., 2006). *T. marneffei* can cause a life-threatening systemic infection in immunocompromised individuals, especially HIV-positive patients (Woo et al., 2012). In recent years, *T. marneffei* has become a leading AIDS-defining diagnosis in Southern Asia, trailing only tuberculosis and cryptococcosis in incidence (Wu et al., 2008; Le et al., 2011; Hien et al., 2016; Lee et al., 2019). Furthermore, *T. marneffei* infection has recently been increasingly observed in HIV-negative adults with no reported immunosuppressive condition, but immunodeficiency is suspected to be the cause of these infections (Ramos-e-Silva et al., 2012; Kauffman et al., 2014). The mortality rate of *T. marneffei* infection exceeds 50% despite antifungal therapy (Le et al., 2011; Hu et al., 2013). Understanding the pathogenic mechanism is fundamental to combating *T. marneffei* infection.

*Talaromyces marneffei* is an intracellular pathogen; conidia are inhaled into a patient’s lungs and subsequently engulfed by alveolar macrophages, where the conidia transform into yeast cells and cause infection (Supparatpinyo et al., 1994). During this process, *T. marneffei* conidia will face a variety of stresses, such as heat, salt stress, oxidative substances, high osmolarity, nutrient deprivation and cytokine-mediated killing (Cao et al., 2009a; Wang et al., 2009; Kummasook et al., 2011). There are several important mechanisms in *T. marneffei* infection, including the conversion of conidia to the yeast phase, resistance to phagocytic killing and oxidative, and heat stress responses (Pongpom et al., 2017), that result in *T. marneffei* survival in macrophages. These strategies are the key processes of immune escape.

In our previous study, we found that the minimal inhibitory concentrations (MICs) of echinocandins were quite low for the *T. marneffei* hyphal form, but *T. marneffei* manifested resistance in its yeast forms (Cao et al., 2009b; Mo et al., 2014). The mechanism by which the *T. marneffei* yeast form is resistant to echinocandins is still unclear, but the cell wall composition is suspected to play a role. Echinocandins are antifungals that inhibit cell wall β-(1,3)-D-glucan synthesis (Douglas et al., 1997). It has been reported that β-(1,3)-D-glucan and chitin are two major components of the fungal cell wall (the other main components are 1,6-β-glucans and mannoproteins) (Klis et al., 2002). Reduced synthesis of β-(1,3)-D-glucan can result in reduced susceptibility to caspofungin, and elevated chitin content can reduce echinocandin efficacy in many fungi (Fortwendel et al., 2009; Cordeiro Rde et al., 2014). In a preliminary study, we found that *T. marneffei* yeast forms were more sensitive to calcium than the hyphal form (Cao et al., 2007). As calcium activates the calcineurin pathway, it is postulated that it may also affect the resistance of *T. marneffei* to echinocandins by regulating cell wall composition and could represent a potential drug target for augmenting echinocandin use in *T. marneffei* infection. Thus, in this study, we aimed to investigate calcineurin function by characterizing the *cnaA* gene and exploring the mechanism of immune escape in *T. marneffei*.

## Materials and Methods

### Strains, Media, and Growth Conditions

Strains used in this study are listed in Table 1. *T. marneffei* FRR2161 is the type strain and was used as the wild-type for all experiments. *T. marneffei* G816 (Δ*ligD niaD^–^ pyrG^–^*) is a uracil/uridine auxotroph (*pyrG*) mutant of FRR2161 (Bugeja et al., 2012). Transformation was performed using the protoplast method (Borneman et al., 2000). The Δ*cnaA* mutant was generated by transforming strain G816 with a linearized Δ*cnaA* deletion construct and selecting for uracil/uridine (*pyrG*^+^) prototrophic transformants. The complemented strain Δ*cnaA cnaA^+^* was generated by transforming the Δ*cnaA* mutant with the *cnaA-ble*-pKB plasmid and selecting for bleomycin (*ble*^+^)-resistant transformants. *T. marneffei* strains were grown at 25°C in *A. nidulans* minimal medium (ANM) with 10 mM (NH_4_)_2_SO_4_ and supplemented appropriately as previously described (Borneman et al., 2000). *T. marneffei* strains were grown at 37°C in BHI medium. *Escherichia coli* DH5ɑ (Invitrogen, United States) was used to clone and propagate the various constructs and was grown in Luria-Bertani broth at 37°C.

**TABLE 1:** Strains and plasmids used in this study.

Strain or plasmid	Genotype or characteristic
*T. marneffei* FRR2161	Wild-type of *T. marneffei*
*T. marneffei* G816	*ligD^–^ niaD^–^ pyrG^–^*
*T. marneffei*Δ*cnaA*	*cnaA^–^ pyrG^+^* of *T. marneffei*
*T. marneffei* Δ*cnaA cnaA^+^*	*cnaA^+^pyrG^–^ ble*^+^ of *T. marneffei*
pBluescript II pSK	Ampicillin-resistant plasmid
*cnaA*-pSK	pBluescript II containing the *cnaA gene*
*pyrG* blaster pSK	Plasmid containing the *A. nidulans pyrG* gene
Δ*cnaA* deletion	Plasmid with *A. nidulans pyrG* gene replacing the *cnaA* coding region
*cnaA-ble-* pKB	Plasmid containing the *cnaA* gene and *ble* resistance gene

To test the radial growth and the spore-producing ability of the mutants, strains were grown in ANM for 14 days at 25°C, and conidia were harvested into sterile Stroke physiological saline solution. A suspension of 1 × 10^5^ conidia per milliliter was prepared. The wild-type, Δ*cnaA* mutant and complemented Δ*cnaA cnaA^+^* strains were inoculated with a 5-μl drop of the 1 × 10^5^ conidia per milliliter suspension onto ANM with 10 mM (NH_4_)_2_SO_4_ with or without uracil, BHI medium or SD medium supplemented with 10 mM (NH_4_)_2_SO_4_. The conidia were incubated at either 25°C or 37°C, and radial growth was measured every day over a period of 14 days. For conidial counts, the number of spores per square millimeter were counted after 14 days at 25°C. The results were analyzed by the *T*-test analysis of variance.

To test for stress responses of the strains, a 5-μl drop of the 1 × 10^5^ conidia per milliliter suspension of each strain was inoculated onto agar-solidified ANM with 10 mM (NH_4_)_2_SO_4_ and 5 mM uracil and supplemented as follows: 0.2, 0.4, 0.6, and 1 M KCl (salt stress); 2, 5 and 8 mM H_2_O_2_(oxidative stress); 0.5, 1, and 1.5 M sorbitol (for osmotic stress); 2.5 and 5 μM Congo red; or 0.1, 5, and 10 μg/ml calcofluor white (cell wall stress). All cultures were incubated for 14 days at 25°C.

### Microscopy

To examine hyphal and yeast cell morphogenesis, conidial germination and cell wall architecture were examined by light microscopy using differential interference contrast (DIC) or epifluorescence optics after staining with calcofluor (CAL). For hyphal growth, conidia were inoculated onto slides covered with a thin layer of agar-solidified ANM with 10 mM (NH4)_2_SO_4_, with or without 5 mM uracil, and incubated at 25°C for 10 days (Borneman et al., 2000). For yeast growth, conidia were inoculated onto slides covered with a thin layer of agar-solidified BHI or SD medium supplemented with (NH_4_)_2_SO_4_ and incubated at 37°C for 10 days.

Ultrastructure analysis was performed using scanning electron microscopy (SEM) and transmission electron microscopy (TEM). For SEM, strains were fixed with 2.5% glutaraldehyde for 2 h at 4°C, washed in 0.1 mol/L phosphate-buffered saline (PBS) three times for 10 min each time and fixed with 1% osmium tetroxide for 1 h. Then, they were washed in 0.1 mol/L PBS three times for 10 min each time and ethanol-dehydrated by sequential washing in 50%, 70%, 80%, 90%, and 100% ethanol. The samples were soaked in hexamethyldisilane three times and dried under vacuum. Thin sections were examined with a Vega 3 LMU-apollo X Scanning Electron Microscope (Tescan, Czechia). For TEM, strains were fixed, washed and dehydrated as described for SEM. The samples were embedded in white resin, and thin sections were examined with a Hitachi H-7650 Transmission Electron Microscope (Hitachi, Japan).

For germination experiments, 10^6^ spores of each strain were inoculated into 30 ml of SD medium supplemented with 10 mM (NH_4_)_2_SO_4_ and incubated for 4, 8, 16 or 24 h at 25°C and 37°C. The rates of germination were determined microscopically by counting the number of germinating conidia in a population of approximately 100 randomly selected spores. Three independent experiments were performed.

### Antifungal Susceptibility Testing

Antifungal susceptibility testing was performed according to the standardized M27-A method approved by the National Committee for Clinical Laboratory Standards (NCCLS) and previously reported methods (Nakai et al., 2003). Caspofungin (CAS), micafungin (MCFG), amphotericin B (AMB), fluconazole (FLC), itraconazole (ITC), and vorconazole (VOC) were purchased from Med Chem Express (New Jersey, NJ, United States). Stock solutions were made with sterile distilled water (MCFG and AMB) or 100% dimethyl sulfoxide (CAS, FLC, ITC and VOC). The stock solutions were diluted in RPMI 1640 medium prepared according to the Clinical and Laboratory Standards Institute (CLSI) standards and then further serially diluted twofold. The final concentration ranges of the antifungals were 0.0625 to 32 μg/ml (CAS and MCFG), 0.0156 to 8 μg/ml (AMB), 0.125 to 64 μg/ml (FLC), and 0.0013 to 1 μg/ml (ITC and VOC). Wild-type and Δ*cnaA* mutant strains were incubated in the presence or absence of drug for 48 h at 25°C and 37°C, and all experiments were performed in triplicate. *Candida parapsilosis* ATCC22019 served as a control.

### Macrophage Assay

RAW264.7 macrophages (1 × 10^5^) (InvivoGen, Hong Kong) were co-incubated with 1 × 10^6^ conidia in DMEM containing 10% fetal bovine serum (Gibco, United States) and 8 mM penicillin-streptomycin at 37°C for 2 h. The cells were then washed with PBS to remove unengulfed conidia and incubated for an additional 24 h at 37°C. Infected macrophages were harvested for microscopy or to determine fungal load. For microscopy, cells were either fixed and prepared for TEM or stained with 5 μM Dil (Invitrogen, United States) for 10 min, washed twice in PBS, fixed in 4% paraformaldehyde, stained with 1 mg/ml calcofluor and examined by light microscopy to observe yeast morphogenesis. For fungal load determination, the number of surviving conidia in macrophages were determined by lysing infected macrophages in cold PBS, diluting 1:100, and plating on YPD medium. After incubating for 72 h at 25°C, the CFU was determined. The test was performed three times in triplicate, and the results were analyzed by *T*-test analysis of variance.

### Murine Model of *Talaromyces marneffei* Infection

Eight-week-old BALB/c mice (male and female) were immunosuppressed with intraperitoneal injections of cyclophosphamide (Sigma-Aldrich) at a dose of 200 mg/kg of body weight on days −4 and −1 and the day of infection, as well as triamcinolone acetonide (Sigma-Aldrich) at a dose of 40 mg/kg of body weight on the day of infection. To evaluate the histopathological progression of disease, four groups of 36 mice were infected with a sublethal dose (10^6^ conidia in 100 μl of physiological saline) of the wild-type, Δ*cnaA*, or Δ*cnaA cnaA^+^* strains or a diluent control (0.9% physiological saline). The mice were sacrificed on days 3, 6, and 9 after inoculation, and their tissue was harvested under sterile conditions. The lung, hepatic and splenic tissues were removed to determine the number of *T. marneffei* by measuring CFU in YPD medium. The test was performed three times in triplicate, and the results were analyzed by *T*-test analysis of variance. To evaluate the mortality rates, four groups of 36 immunosuppressed mice were challenged with 100 μl of suspensions containing 10^8^ conidia/ml of each strain. The morbidity and mortality of the mice were observed every day for 14 days. Survival was plotted on a Kaplan-Meier curve, and a log-rank test was used for pairwise comparisons among the strains.

## Results

### Calcineurin Genes in *Talaromyces marneffei*

The sequence of the *T. marneffei cnaA* gene was obtained from GenBank (GenBank accession no. XM_002147834.1, ATCC18224). The gene encompasses 2261 bp and encodes a putative gene product of 557 amino acids. In searches against the GenBank database, the *T. marneffei cnaA* gene showed strong homology to the sequences in *Talaromyces stipitatus* (XM_002482031.1, 88% identity), *Aspergillus aculeatus* (XM_020205250.1, 79% identity), and *Aspergillus nomius* (XM_015546462.1, 78% identity).

### Loss of *cnaA* Affects Colonial Morphology and Radial Growth

**FIGURE 1:**
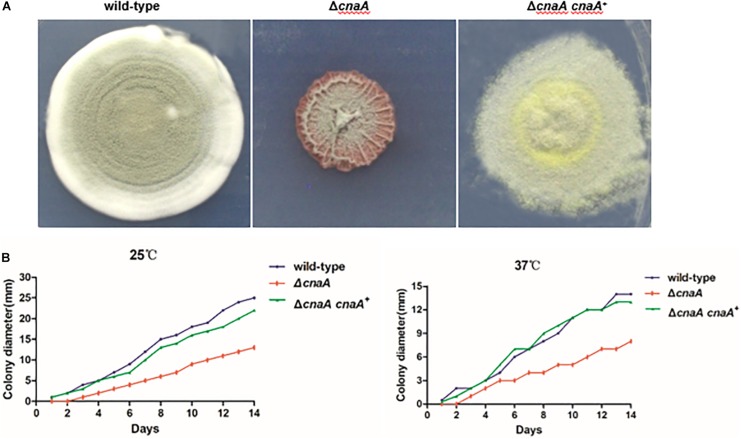
Loss of *cnaA* affects colonial morphology and radial growth. **(A)** *T. marneffei* wild-type and Δ*cnaA cnaA^+^* strains exhibited highly vegetative hyphae that appeared fluffy and green and developed many conidiophores at 25°C when grown on ANM. The colony edge was relatively uniform and compact. Colonies of the Δ*cnaA* mutant showed thin growth and exhibited decreased aerial hyphae production, resulting in a film-like surface morphology that was wrinkled. **(B)** Conidia from each strain were inoculated on ANM or BHI medium and incubated for 1–14 days at 25°C and 37°C. The Δ*cnaA* mutant grew less than the wild-type and Δ*cnaA cnaA^+^* complemented strain.

Wild-type *T. marneffei* growing at 25°C produces colonies comprised of vegetative hyphae that appear fluffy around the periphery and green in the center due to asexual development (conidiation) and the production of pigmented conidia. The colony edge is relatively uniform and compact. In contrast, colonies of the Δ*cnaA* mutant exhibited a significant reduction in radial growth rate with sparse growth and low aerial hyphae production. The colony surface was wrinkled, and the periphery was irregular. The colony also produced more of the red pigment that is characteristic of *T. marneffei* (Figure 1A). The Δ*cnaA* mutant colonies also readily detached from the medium, indicating a lack of invasive growth. The complemented strain (Δ*cnaA cnaA*^+^) displayed colony phenotypes that were very similar to that of the wild-type.

The radial growth rate of the wild-type, Δ*cnaA* and Δ*cnaA cnaA*^+^ strains was quantified during growth on solid medium at both 25°C and 37°C. At 25°C, the wild-type and Δ*cnaA cnaA*^+^ strains showed similar growth rates, while that of the Δ*cnaA* mutant was substantially reduced. Similarly, at 37°C, the wild-type and Δ*cnaA cnaA^+^* strains showed similar growth rates, while that of the Δ*cnaA* mutant was reduced (Figure 1B).

### Loss of *cnaA* Affects Conidiophore Development and Conidial Germination

**FIGURE 2:**
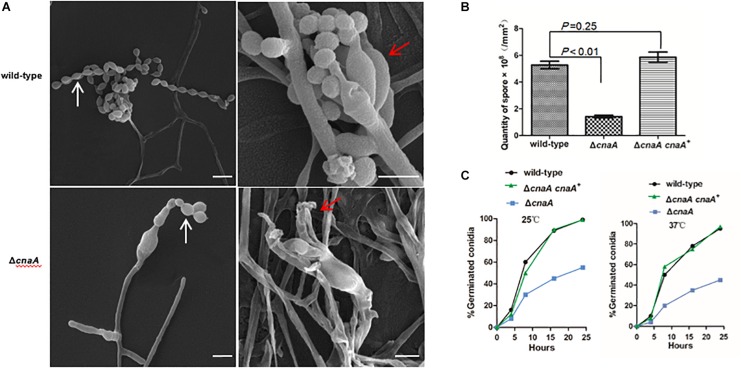
Loss of *cnaA* affects conidiophore development and conidial germination. **(A)** Wild-type and Δ*cnaA* strains were grown on ANM + (NH_4_)_2_SO_4_ for 14 days at 25°C and examined by SEM. The wild-type strain showed a conidiophore that was composed of four to seven phialides (red arrow) and long chains of conidia (white arrow), resulting in the production of abundant conidia. In the Δ*cnaA* mutant, the conidiophore displayed abnormal phialide and spore chains with decreased numbers of conidia. Scale bars, 10 μm. **(B)** Compared with the wild-type and Δ*cnaA cnaA^+^* complemented strain, the Δ*cnaA* mutant showed a statistically significant decrease in conidial density (*P* < 0.01). **(C)** The kinetics of germination were measured at both 25°C and 37°C by counting the number of germinating conidia in a population of approximately 100 spores after incubation for 4, 8, 16, and 24 h in SD liquid medium. In contrast to the wild-type, the percentage of germination decreased in the Δ*cnaA* mutant.

To examine the cellular basis of the poor conidiation observed for the Δ*cnaA* mutant, the various strains were examined by SEM. After 14 days of incubation at 25°C, the wild-type showed conidiophores composed of a stalk cell bearing four to seven phialides, and each phialide had many conidia arranged in a chain. The wild-type conidial chains were very long and resulted in the production of abundant conidia. In contrast, conidiophore integrity was aberrant and conidiation was reduced in the Δ*cnaA* mutant. Phialides of the Δ*cnaA* mutant were abnormal in morphology, and their number was highly reduced compared with that of the wild-type strain (Figure 2A). Compared with the wild-type strain, the Δ*cnaA* mutant showed a statistically significant decrease in conidial density (*P* < 0.01), which directly correlates with the reduced phialide numbers. The Δ*cnaA cnaA^+^* complemented strain was identical to the wild-type (Figure 2B).

The kinetics of germination were measured by counting the number of germinating conidia in a population of approximately 100 conidia after incubation for 4, 8, 16, and 24 h in Sabouraud’s (SD) liquid medium at either 25°C and 37°C. In contrast to the wild-type and Δ*cnaA cnaA^+^* strains, the Δ*cnaA* strain show a visible delayed germination at 25°C and 37°C. In the first 4 h, a steady rate of germination was evident for both the wild-type and complemented strains, which was comparable to that of the Δ*cnaA* mutant, although the Δ*cnaA* mutant at 37°C started to show a decrease in germination compared with that at 25°C. This difference was accentuated at the longer time points and became evident by 8 h. By 24 h, the Δ*cnaA* mutant exhibited a germination percentage that was half that of the wild-type and complemented strains, which may be one reason why the mutant exhibited a delay in growth at longer incubation time points (Figure 2C).

### Δ*cnaA* Mutant Displays Defects in Hyphal and Yeast Morphogenesis

**FIGURE 3:**
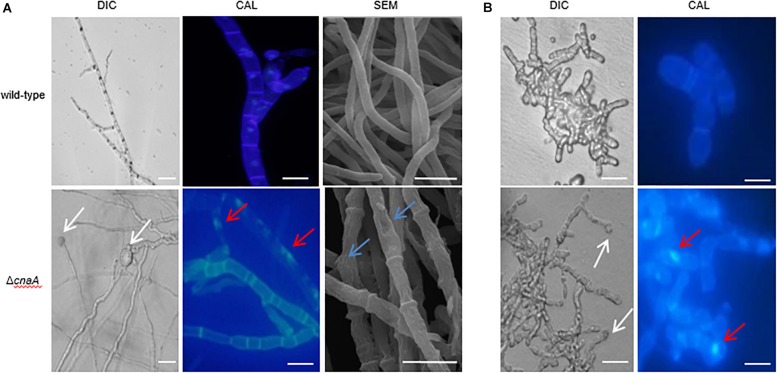
The *cnaA* mutant displays defects in hyphal and yeast morphogenesis. **(A)** Wild-type and Δ*cnaA* strains were inoculated onto ANM medium and grown at 25°C for 10 days, followed by either staining with calcofluor to visualize cell walls by light microscopy or SEM. The wild-type hyphal cells showed a smooth and uniform hyphal diameter with regular septation and uniform staining with calcofluor white. In contrast, the Δ*cnaA* mutant showed irregular hyphal growth with enlarged cells (white arrow) and exhibited abnormal chitin deposits along the hyphae (red arrow). SEM images show that the mutant hyphal cells appear shrunken and irregularly shaped with very prominent septa. **(B)** The terminal ends of yeast cells of the Δ*cnaA* mutant were swollen (white arrow) and exhibited abnormal chitin deposits (red arrow). Scale bars, 10 μm.

To examine the cellular basis for the observed macroscopic growth defects observed in the Δ*cnaA* strain, all strains were grown on ANM or BHI medium for 10 days at 25°C and 37°C and either stained with calcofluor white (CAL) to visualize the cell walls by fluorescence microscopy or processed for SEM. The wild-type hyphal cells showed a smooth and uniform hyphal diameter with regular septation and uniform staining with CAL. In contrast, the Δ*cnaA* mutant exhibited irregularly shaped hyphal cells that were enlarged in diameter, particularly at septation sites, and showed abnormal CAL-stained chitin deposits along the hyphae. This result suggests that calcineurin is important for proper hyphal extension (Figure 3A). Compared with the wild-type, the poles of the yeast cell of the Δ*cnaA* mutant showed abnormal swelling and exhibited abnormal chitin deposits. Therefore, the *cnaA* mutant displayed defects in yeast morphogenesis (Figure 3B).

### *cnaA* Is Required for Correct Cell Wall Biosynthesis in *Talaromyces marneffei*

**FIGURE 4:**
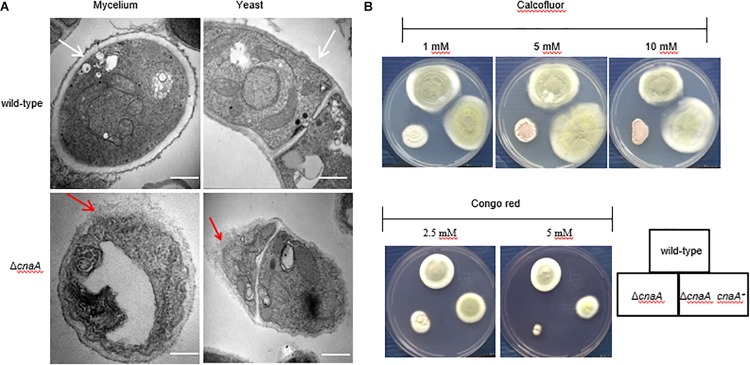
The *cnaA* gene is required for correct cell wall biosynthesis. **(A)** The morphology of the strains was observed by TEM after 10 days of growth in ANM medium at 25°C and 37°C. The cell wall of the wild-type strain was smooth and intact in the hyphal and yeast forms *in vitro*, and the organelles were clearly visible (white arrow). In contrast, the Δ*cnaA* mutant showed cell wall deformation with less distinct layering, and the organelles of the mutant appeared disordered (red arrow). Scale bars, 0.2 μm. **(B)** Strains were grown on ANM medium at 25°C after the addition of various concentrations of calcofluor white or Congo red. Compared with the wild-type, the Δ*cnaA* mutant showed greater sensitivity to Congo red at concentrations of 5 mM, but there was no difference in calcofluor white staining.

The hyphal and yeast morphogenesis defects noted for the Δ*cnaA* strain were examined further by TEM. The strains were grown on agar-solidified ANM and BHI medium for 10 days at 25°C and 37°C and processed for TEM. For the wild-type strain, transverse sectioning of the hyphal cells showed a cell wall composed of three layers, namely, a thin inner membrane-proximal layer that was electron-dense, a thick middle layer that was electron-transparent and an irregular outer layer with protrusions. The wild-type yeast cells showed a similar cell wall architecture but with a smoother outer wall layer. In both cell types, the organelles could also be clearly seen. In contrast, the Δ*cnaA* mutant displayed cell wall perturbations in all of the layers for both hyphal and yeast cells, and the organelles appeared less distinct (Figure 4A).

The observed changes in the cell wall architecture were further examined by testing the sensitivity of the various strains to cell wall-perturbing agents and a variety of antifungal drugs. Compared with the wild-type strain, the Δ*cnaA* mutant was more sensitive to Congo red at 5 mM and 25°C but equally sensitive to different CAL white concentrations (Figure 4B). When tested for sensitivity to various anti-fungal agents, the MICs for caspofungin (CAS) and micafungin (MCFG) for the mycelial form were much lower than those for the yeast form in the parental strain of *T. marneffei*. In contrast to the parental strain, potent activity against the yeast form of the Δ*cnaA* mutant was observed. The MIC of CAS was reduced from 16 μg/ml (wild-type) to 2 μg/ml (mutant), and the MIC of MCFG was similarly reduced from 32 μg/ml to 4 μg/ml at 37°C. When testing azole-based antifungals, the MICs of fluconazole (FLC), itraconazole (ITC), and voriconazole (VOC) for the mutant were a gradient lower than the corresponding MICs for the wild-type strain (Table 2). All of the above evidence suggests that the *cnaA* gene participates in cell wall integrity in a direct manner.

**TABLE 2:** *In vitro* assay of antifungal susceptibility.

Antifungal drugs	Hyphal MIC (μg/ml)		Yeast MIC (μg/ml)	
	wild-type	Δ*cnaA*	wild-type	Δ*cnaA*
Amphotericin B	0.5	0.5	0.5	0.5
Itraconazole	8	8	8	4
Fluconazole	0.03	0.03	0.03	0.01
Voriconazole	0.06	0.03	0.06	0.03
Caspofungin	2	0.5	16	2
Micafungin	4	2	32	4

### *cnaA* Is Required for *Talaromyces marneffei* Adaptation to High Osmolarity *in vitro*

**FIGURE 5:**
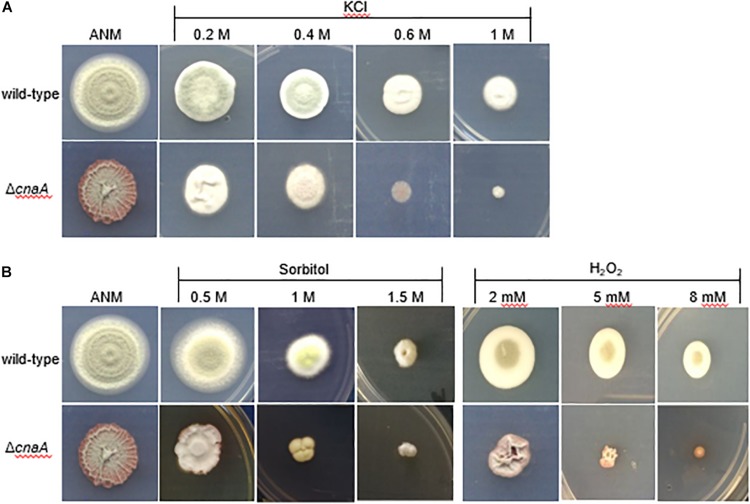
The *cnaA* gene is required for adaptation to osmotic stress *in vitro.* To test the stress resistance of mutants, wild-type, and Δ*cnaA* strains were inoculated with a 5-μl drop of a 1 × 10 ^5^ conidia/ml suspension onto ANM supplemented as follows: **(A)** for salt stress using 0.2, 0.4, 0.6, and 1M KCl; **(B)** for oxidative stress adding 2, 5 and 8 mM H_2_O_2_; and for osmotic stress adding 0.5, 1, and 1.5 M sorbitol, followed by incubation for 14 days at 25°C. The Δ*cnaA* mutant showed a gradual reduction in growth with increasing concentrations of the stress agent, especially for oxidative stress.

To investigate whether the Δ*cnaA* mutant of *T. marneffei* exhibits sensitivity to salt stress, oxidative stress and high osmolarity conditions, the Δ*cnaA* mutant and parental strain were incubated on ANM containing different concentrations of potassium chloride (KCl), H_2_O_2_, or sorbitol. The results revealed that the Δ*cnaA* mutant was highly sensitive to oxidative stress. Compared with the parental strain, the Δ*cnaA* mutant exhibited gradually reduced growth in increasing KCl concentrations, and the growth inhibition was more obvious at high concentrations (0.6 M, 1 M KCl) than at low concentrations (Figure 5A). The growth of the Δ*cnaA* mutant was similar to that of the parental strain at low concentrations of sorbitol, but mutant growth inhibition was obvious in 1M and 1.5 M sorbitol. In contrast to the parental strain, the Δ*cnaA* mutant was highly sensitive to oxidative stress; the growth of the mutant was inhibited in 5 and 8 mM H_2_O_2_ (Figure 5B).

### *cnaA* Is Essential for Immune Escape in Macrophages

**FIGURE 6:**
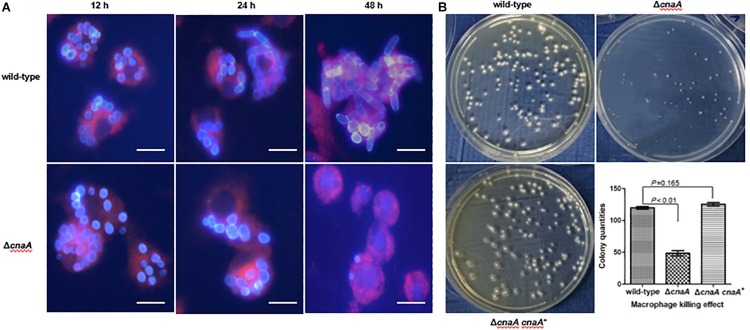
The *cnaA* gene is essential for immune escape in macrophages. **(A)** Wild-type and Δ*cnaA* strains were co-cultured with RAW264.7 macrophages and observed by confocal microscopy. After 12, 24, and 48 h following phagocytosis, the Δ*cnaA* mutant conidia showed increased sensitivity to the cytotoxic activity of the macrophages and were killed and eliminated, compared with the wild-type, which germinated into yeast cells and replicated profusely intracellularly. Scale bars, 20 μm. **(B)** Conidial survival was measured by counting CFU on SD medium after lysis of *T. marneffei*-infected macrophages. The number of surviving Δ*cnaA* mutant cells was drastically lower than the number of surviving wild-type cells (*P* < 0.01).

**FIGURE 7:**
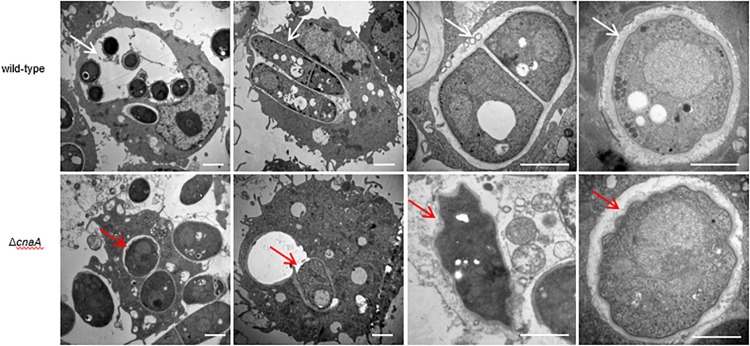
The *cnaA* gene is essential for survival in macrophages. Wild-type and Δ*cnaA* strains were co-cultured with RAW264.7 macrophages and observed by TEM. Wild-type conidia germinated into yeast cells and maintained cellular integrity and morphology after 24 h. The septum of dividing cells is clearly visible, the cell wall is complete and uniform, and organelles are distinct (white arrow). In contrast, most of the Δ*cnaA* mutant conidia failed to germinate or were clearly atrophic with little to no organellar integrity (red arrow). Scale bars, 2 μm.

To assess whether the observed changes in the cell wall architecture and sensitivity to a broad range of stressors in the Δ*cnaA* strains affected its ability to interact with host macrophages, the various strains were co-incubated with RAW264.7 macrophages and examined after 12, 24, and 48 h of incubation. The wild-type conidia co-incubated with RAW264.7 macrophages were rapidly phagocytosed and germinated into ellipsoid-shaped yeast cells. By 24 h, these yeast cells had grown in size and were dividing by fission. This continued to 48 h, where macrophages were filled with dividing yeast cells. Conidia from the Δ*cnaA* strain were also readily phagocytosed by RAW264.7 macrophages, and by 12 h, the conidia showed clear signs of germination into ellipsoid-shaped yeast cells, similar to the wild-type. However, by 24 h, the Δ*cnaA* yeast cells showed much slower growth than the wild-type cells, with only minimal signs of division. At 48 h, there were very few yeast cells remaining in macrophages, showing that most of the yeast cells had been killed and degraded (Figure 6A). TEM observations clearly showed that wild-type cells maintained normal cellular morphology and developed into yeast cells in macrophages. The septum was visible, the cell wall of yeast cells was complete and uniform, organelles were clear and the cytoplasm was uniform. However, most of the Δ*cnaA* conidia were atrophic and destroyed by the phagosome (Figure 7).

Conidia survival was measured by lysing a fixed number of macrophages infected with either the wild-type or Δ*cnaA* strain after co-incubation for 24 h and counting viable *T. marneffei* cells (colony forming units, CFU) on YPD medium after 72 h of incubation at 25°C. The number of surviving Δ*cnaA* mutant cells was drastically lower than the number of surviving wild-type cells, and over 60% of the Δ*cnaA* cells were killed at this time point (*P* < 0.01) (Figure 6B). Therefore, *cnaA* plays an important role in resisting macrophage killing.

### Loss of *cnaA* Abrogates Virulence in a Murine Model of Invasive *Talaromyces marneffei* Infection

**FIGURE 8:**
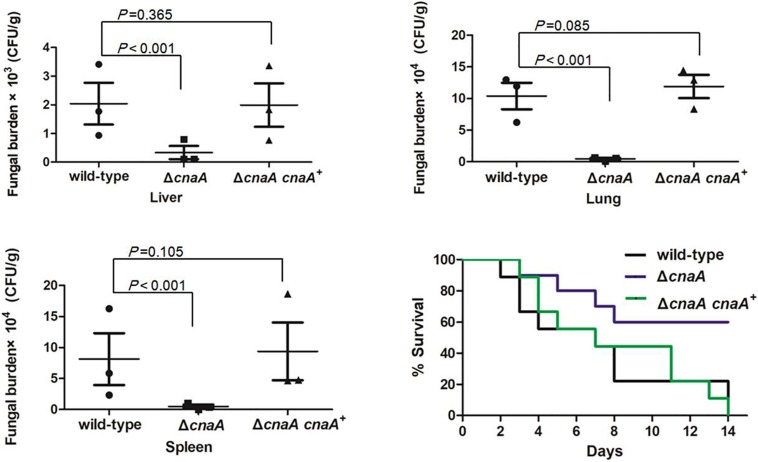
Loss of *cnaA* abrogates virulence in a murine model of invasive *T. marneffei* infection. Mice were infected with 10^6^ conidia from the wild-type, Δ*cnaA* or Δ*cnaA cnaA*^+^ strains by intraperitoneal injection. Organs were harvested 3, 6, or 9 days after infection, and 3 mice were sacrificed every day. Tissue was then assayed for viable *T. marenffei*. Compared with that for the wild-type and Δ*cnaA cnaA*^+^ strains, the number of CFU for the Δ*cnaA* mutant was significantly reduced in lung, hepatic and splenic tissue. The results were statistically significant based on *T*-test analysis of variance (*P* < 0.001) for all comparisons. The morbidity and mortality of infected mice were assessed every day for 14 days. Survival was plotted on a Kaplan-Meier curve and showed that mice infected with the Δ*cnaA* mutant had a reduced fatality rate (45%) at 14 days compared with the wild-type and Δ*cnaA cnaA*^+^ strains.

To examine the role of *cnaA* in virulence, a murine model of *T. marneffei* infection was utilized to mimic human disease. Four groups of 36 mice were infected by intraperitoneal injection with a sublethal dose (10^6^ conidia in 100 μl of physiological saline) of the wild-type, Δ*cnaA*, Δ*cnaA* and *cnaA*^+^ strains and a diluent control (0.9% physiological saline). The mice were sacrificed on days 3, 6, and 9 postinfection, and their tissues (lung, liver, and spleen) were harvested under sterile conditions. These tissues were macerated, plated on YPD medium and incubated at 25°C for 72 h for CFU assessment. Compared with the control group, the Δ*cnaA* mutant group exhibited sharply decreased CFU in the lung, hepatic, and splenic tissues (*P* < 0.001) (Figure 8). Infected mice displayed severe signs of invasive disease, including hunched posture, shivering, ruffled fur and emaciation. To evaluate the mortality rates, four groups of 36 immunosuppressed mice were challenged with 100 μl of suspensions containing 10^8^ conidia/ml of each strain. The mortality rates of the mice infected with either the wild-type or Δ*cnaA cnaA^+^* strains were similar, and these mice all died by 14 days postinfection. In contrast, the mortality rate of the Δ*cnaA* mutant-infected mice was 45% at 14 days (Figure 8). These data clearly demonstrate that *cnaA* affects virulence in a murine model of invasive *T. marneffei* infection.

## Discussion

This study investigated the roles of a calcineurin homolog (*cnaA*) in the dimorphism and pathogenicity of the opportunistic human fungal pathogen *T. marneffei*. We have shown that *cnaA* (i) is necessary for conidiation, germination, hyphal and yeast cell morphogenesis and growth; (ii) plays an essential role in cell wall integrity of both hyphal and yeast cell types; (iii) is required for stress adaptation for hyphal and yeast cell types; (iv) plays a unique role during immune escape; and (v) is required for full virulence in a murine model of invasive *T. marneffei* infection.

Conidia are an important cell type for almost all fungi and are often the infectious propagules of pathogenic fungi. Their production is tightly regulated, as is their capacity to sense the environment and germinate to initiate vegetative growth. Thus, conidiation and germination are central aspects of fungal cell survival and propagation and important pathogenicity determinants (Boyce and Andrianopoulos, 2007). Infection by *T. marneffei* is believed to occur by inhalation of conidia into the lungs, where they subsequently germinate and transform into yeast cells that cause disseminated infection. Deletion of the *cnaA* gene severely affected asexual reproduction, with the mutant showing defects in the development of the conidiophore, and this resulted in a sharp decrease in the number of conidia produced. It has been reported that calcineurin controls conidiation in *Aspergillus fumigatus* (Shwab et al., 2019), *Aspergillus nidulans* (Wang et al., 2012), and *Penicillium digitatum* (Zhang et al., 2013). In addition, a Δ*cnaA* mutant in *Beauveria bassiana* has been shown to have differential defects in conidial germination, vegetative growth and conidiation capacity (Huang et al., 2015; Wang et al., 2017). These findings show that *cnaA* plays important roles in both production of conidia and their ability to convert to vegetatively growing cells, both of which are likely to affect invasive infection in *T. marneffei*.

The cell wall is a physically rigid, yet plastic, structure that is responsible for the shape of the cell, protects the fungal cell from its environment, prevents killing by predators and mediates cell-cell interaction (Fontaine et al., 2000). Fungal cell walls are unique, and cell wall carbohydrates and proteins play important roles in cell physiology and disease pathogenesis (Mancuso et al., 2018). In *Candida tropicalis*, it has been shown that calcineurin is essential for tolerance of azoles, caspofungin, anidulafungin, and cell wall-perturbing agents (Chen et al., 2014). Similarly, in *Cryptococcus neoformans*, caspofungin tolerance is mediated by multiple pathways downstream of calcineurin function (Pianalto et al., 2019). This study showed that the *T. marneffei*Δ*cnaA* mutant displays defects in the hyphal and the yeast cell wall and that the *cnaA* gene is important for cell wall integrity. This was supported by the observation that yeast cells of the Δ*cnaA* mutant showed a lower MIC against caspofungin (CAS; eightfold) and micafungin (MCFG; eightfold) than wild-type cells (Table 2). CAS and MCFG are members of the echinocandin class of antifungal agents that inhibit fungal cell wall biosynthesis by inhibiting cell wall β-(1,3)-D glucan synthesis (Douglas et al., 1997). These results suggest that targeting calcineurin in combination with echinocandin treatment may be effective in *T. marneffei* infection, whereas echinocandins on their own are not.

The first line of defense in the human body against *T. marneffei* infection is the innate immune system (Romani, 2011). For *T. marneffei*, initial interactions are characterized by phagocytosis of the conidia by leukocytes in the lungs, followed by leukocyte-facilitated hematogenous dissemination (Vanittanakom et al., 2006). *T. marneffei* conidia face a variety of stresses, such as heat, salt, oxidative stress, osmolarity, nutrient deprivation and cytokine-mediated killing (Pongpom et al., 2017; Ellett et al., 2018). *T. marneffei* shows strong stress tolerance and the ability to resist the cytotoxicity of macrophages in the innate immune system (Pongpom et al., 2005; Vanittanakom et al., 2009). The Δ*cnaA* mutant showed increased sensitivity to salt, H_2_O_2_ and osmotic stress *in vitro* during hyphal growth. It has been reported that calcineurin is essential in stress resistance in *C. albicans* (Reedy et al., 2010; Liu et al., 2014). This stress adaptation not only helps *T. marneffei* survive in extreme environments but also plays important roles in resisting killing and replication inside macrophages. In fact, morphogenesis and survival of the Δ*cnaA* yeast was compromised inside host cells, but the mutant cells were still able to germinate and develop *in vitro*. During macrophage infection, the Δ*cnaA* mutant conidia hardly germinated or underwent yeast morphogenesis after being phagocytosed, and at longer incubation times, these conidia were killed and eliminated by the macrophages. Thus, the Δ*cnaA* mutant is defective in resisting killing by macrophages.

The phenotypes of the Δ*cnaA* mutant suggested that it would likely be compromised in virulence, and we showed that the deletion of *cnaA* resulted in a drastic increase in the mean survival time of systemically infected mice, with a substantially reduced fungal burden in the lung, hepatic and splenic tissues compared with the wild-type-infected mice. These results indicated that *T. marneffei cnaA* affects virulence in the murine model of invasive *T. marneffei* infection and that it is important for full virulence but does not block infection and dissemination. The capacity for the *T. marneffei*Δ*cnaA* mutant to disseminate despite showing severely compromised survival in an *in vitro* macrophage assay may suggest that there are additional routes of dissemination in an animal host. The importance of calcineurin in virulence has also been shown in *A. fumigatus*, *C. neoformans*, and *C. tropicalis* (Fox et al., 2001; Chen et al., 2014; Juvvadi et al., 2014).

In summary, our findings show that c*naA*, and therefore calcineurin, plays a key role in controlling fungal morphogenesis and the response of *T. marneffei* to external stresses, including antifungal drugs as well as the host immune response and subsequent fungal pathogenicity. It is required for full virulence in a murine model of invasive *T. marneffei* infection. Moreover, *cnaA* could be a potential target for combinatorial antifungal therapy during life-threatening *T. marneffei* systemic infections.

## Data Availability Statement

All datasets generated for this study are included in the article/supplementary material.

## Ethics Statement

This study protocol was approved by the Ethics Committee of the First Affiliated Hospital of Guangxi Medical University (ethics amendment dated 4/3/2012, approval number KY-074). All experiments in this study were conducted according to internationally accepted standards and regulations on the administration of experimental animals in China (8/1/2011 C-WISC).

## Author Contributions

C-WC designed this study and drafted the manuscript. Y-QZ and K-SP performed the experiment and data analysis. Y-QZ calculated the statistics and edited the manuscript. J-PL and AA provided the valuable advice, supported the experiment protocol, and critically revised the manuscript. AA critically revised the manuscript. HL, R-FY, J-YW, and C-YH assisted in completing the experiment. All authors have read and approved the final manuscript.

## Conflict of Interest

The authors declare that the research was conducted in the absence of any commercial or financial relationships that could be construed as a potential conflict of interest.
